# Detection of Phosphatidylcholine-Coated Gold Nanoparticles in Orthotopic Pancreatic Adenocarcinoma using Hyperspectral Imaging

**DOI:** 10.1371/journal.pone.0129172

**Published:** 2015-06-05

**Authors:** Christopher G. England, Justin S. Huang, Kurtis T. James, Guandong Zhang, André M. Gobin, Hermann B. Frieboes

**Affiliations:** 1 Department of Pharmacology & Toxicology, University of Louisville, Louisville, KY, United States of America; 2 James Graham Brown Cancer Center, University of Louisville, Louisville, KY, United States of America; 3 Department of Medicine, University of Louisville, Louisville, KY, United States of America; 4 Department of Bioengineering, University of Louisville, Louisville, KY, United States of America; The Liverpool Cancer Research UK Centre, UNITED KINGDOM

## Abstract

Nanoparticle uptake and distribution to solid tumors are limited by reticuloendothelial system systemic filtering and transport limitations induced by irregular intra-tumoral vascularization. Although vascular enhanced permeability and retention can aid targeting, high interstitial fluid pressure and dense extracellular matrix may hinder local penetration. Extravascular diffusivity depends upon nanoparticle size, surface modifications, and tissue vascularization. Gold nanoparticles functionalized with biologically-compatible layers may achieve improved uptake and distribution while enabling cytotoxicity through synergistic combination of chemotherapy and thermal ablation. Evaluation of nanoparticle uptake *in vivo* remains difficult, as detection methods are limited. We employ hyperspectral imaging of histology sections to analyze uptake and distribution of phosphatidylcholine-coated citrate gold nanoparticles (CGN) and silica-gold nanoshells (SGN) after tail-vein injection in mice bearing orthotopic pancreatic adenocarcinoma. For CGN, the liver and tumor showed 26.5±8.2 and 23.3±4.1 particles/100μm^2^ within 10μm from the nearest source and few nanoparticles beyond 50μm, respectively. The spleen had 35.5±9.3 particles/100μm^2^ within 10μm with penetration also limited to 50μm. For SGN, the liver showed 31.1±4.1 particles/100μm^2^ within 10μm of the nearest source with penetration hindered beyond 30μm. The spleen and tumor showed uptake of 22.1±6.2 and 15.8±6.1 particles/100μm^2^ within 10μm, respectively, with penetration similarly hindered. CGH average concentration (nanoparticles/μm^2^) was 1.09±0.14 in the liver, 0.74±0.12 in the spleen, and 0.43±0.07 in the tumor. SGN average concentration (nanoparticles/μm^2^) was 0.43±0.07 in the liver, 0.30±0.06 in the spleen, and 0.20±0.04 in the tumor. Hyperspectral imaging of histology sections enables analysis of phosphatidylcholine-coated gold-based nanoparticles in pancreatic tumors with the goal to improve nanotherapeutic efficacy.

## Introduction

Systemic delivery of nanoparticles to solid tumors can be considered in three distinct stages: systemic travel while avoiding sequestering by the reticuloendothelial system (RES, mainly the liver and the spleen), extravasation from intra-tumoral capillaries, and diffusion to reach and penetrate malignant cells [1, 2]. The compromise of any of the associated processes can lead to suboptimal nanoparticle uptake within tumor tissue, resulting in diminished therapeutic or diagnostic efficacy. To facilitate systemic travel, surface modifications have been applied to nanoparticles to enhance passive and active targeting while minimizing RES sequestering, e.g., coating with the hydrophilic polymer poly(ethylene)-glycol (PEG) results in prolonged circulation time and diminished RES uptake [3, 4]. Coating with targeting ligands specific to over-expressed receptors on cancer cells (e.g., high-density lipoprotein (HDL) receptor) can further enhance nanoparticle uptake and accumulation at the tumor site [5].

However, the typically irregular tumor vasculature resulting from uncoordinated pro- and anti-angiogenic stimuli further hinders nanoparticle passive transport to cellular targets as well as therapeutic efficacy even if systemic travel and targeted accumulation were successful [6]. Hypo-vascularized regions of tissue lead to cells invoking survival mechanisms to overcome oxygen and nutrient deprivation including minimization of metabolism (quiescence) resulting in cell cycle-dependent chemotherapeutic resistance [7]. In addition to intrinsic and microenvironment-associated resistance mechanisms, cells distant from vasculature in solid tumors receive suboptimal levels of diffusible substances as transport may be limited by increased interstitial fluid pressure (IFP), a chaotic and dense extracellular matrix, and an acidic microenvironment [6, 8–11].

To overcome these transport barriers and successfully deliver nanoparticles more homogeneously into tumor tissue, nanoparticle size and surface modifications have traditionally been modulated [12]. Studies have described positive correlations between 50 nm nanoparticles and enhanced tumor accumulation and diffusivity within tumor tissue [13], while larger 100 nm nanoparticles are readily filtered by the liver and their transport is hindered once at the tumor due to a dense extracellular matrix (ECM) [14, 15]. Very small (5 nm) gold nanoparticles conjugated with anti-EGFR antibody were shown to successfully target and treat pancreatic cancer when injected intraperitoneally into mice [16]. Using 3D cell cultures, size-specific localization of nanoparticles was demonstrated by showing that small nanoparticles (2 and 6 nm) exhibited superior penetration in comparison to slightly larger (15 nm) nanoparticles [17]. It was also shown that nanoparticles ~20 nm displayed superior penetration in comparison to even larger nanoparticles [15]. Previously, we have shown that smaller citrate gold nanoparticles (45–60 nm) display enhanced tissue diffusivity in comparison to larger silica-gold nanoshells (160–175 nm) [18]. We also evaluated the effects of surface modifications on passive transport by functionalizing nanoparticles with phosphatidylcholine (PC) and high-density lipoprotein (HDL). Results showed that both PC-coated and HDL-coated nanoparticles displayed enhanced tissue penetration compared to PEGylated nanoparticles in 3D cell cultures of human pancreatic, lung, and hepatocellular cancers [18]. As PEGylation is the most common surface modification, the results demonstrated that such alternative surface modifications might enhance passive transport through solid tumor tissue.

Nanoparticle localization is usually assessed using scanning electron microscopy (SEM) and transmission electron microscopy (TEM), yet these methods alone cannot conclusively detect nanoparticles. In combination with other tools (e.g., energy dispersive X-ray (EDX) microanalysis), elemental analysis can provide further confirmation of nanoparticle identity [19]. Here, we employ hyperspectral imaging to create libraries of known nanoparticles and thus enable their detection within tissue samples or individual cells. Recently, the deposition patterns of cisplatin aerosol therapy in surgically resected stage II lymph nodes from lung cancer patients was analyzed using hyperspectral imaging [20]. Further, recombinant human epidermal growth factor was encapsulated into liposomes, from which the morphology and particle distribution was analyzed using hyperspectral imaging [21]. This imaging has also been utilized in patients for rapid, on-sight histological classification of lung cancer [22], demonstrating the flexibility of this technique in biomedical applications. Although nanoparticles can be identified using dark field microscopy alone, the combination of hyperspectral imaging with dark field microscopy facilitates automatic detection while minimizing measurement error.

We examine the uptake and diffusivity of PC-coated citrate gold nanoparticles and silica-gold nanoshells in tumors of the pancreas. We focus on pancreatic cancer as it is the fourth most common cause of cancer-related death in the United States, with dismal 5-year relative survival rates <6% [23]. Due to the late onset of clinical symptoms and a paucity of known biomarker candidates, pancreatic cancer prevention and diagnosis remain difficult, with over 70% of pancreatic cancer cases diagnosed at stage III or IV, when surgical intervention is generally no longer an option as the disease has spread through metastasis [24]. Current treatment options fail to effectively cure pancreatic tumors and have only provided minimal increases in survival rates, demonstrating the vital need for novel treatment options. Here, we synthesize and characterize PC-coated gold nanoparticles using methods outlined previously [18], and tail-vein inject them into mice bearing orthotopic pancreatic tumors. This allows for more realistic evaluation of nanoparticle uptake and delivery than in subcutaneous models. After allowing for 48 hours of circulation, the animals were euthanized and the organs harvested for histological analysis. *Ex vivo* identification and localization of nanoparticles in the liver, spleen, and pancreatic tumor tissues were determined using hyperspectral imaging of histology sections, and independently assessed using silver enhancement staining. Results were compared to previous work in which uptake of these nanoparticles was assessed in 3D cell culture [18]. This work suggests that hyperspectral imaging of tissue histology sections may help to detect phosphatidylcholine-coated gold nanoparticles in orthotopic pancreatic tumors.

## Materials and Methods

### Synthesis of Citrate Gold Nanoparticles

As previously described [18], nanoparticles were synthesized using the method in which gold chloroauric acid is reduced by trisodium citrate [25]. In this process, 2.2–2.4 mL 1% weight/volume (wt/v) sodium citrate (Fisher Scientific, Waltham, MA, USA) and 200 mL 0.01% wt/v HAuCl4 (Alfa Aesar, Ward Hill, MA, USA) are mixed and heated to boiling, which promotes the reaction of sodium citrate to citric acid. Temperature and final concentration of the gold salt allows particles of varying sizes. Once the reaction is completed, the solution is concentrated using a rotovapor (Buchi Rotovapor System, BÜCHI Labortechnik AG, Flawil, Switzerland) to ~20 mL before the addition of layering to the particles.

### Synthesis of Silica Gold Nanoshells

Particles have an inner core composed of silica with an outer coating of gold. Synthesis consists of four stages: production of a colloid of small gold particles (2–4 nm) through reduction and aging of gold colloid produced by the recipe of Vogel et al. [26], fabrication of monodispersed silica cores from the Stöber method [27, 28], attachment the seeds to the silica surface, and finally gold shell growth via reduction of additional gold. The gold colloid solution is created utilizing the THPC (Tetrakis(hydroxymethyl)phosphonium chloride) method [29]. Growth of the silica cores requires the combination of 7.5 mL tetraethyl-orthosilicate (TEOS, Sigma Aldrich, St Louis, MO, USA), 225 mL absolute ethanol (Decon Labs, King of Prussia, PA, USA), and 12.5–13.5 mL ammonia (Sigma Aldrich). Ammonia is adjusted to achieve silica core sizes 110 ± 5 nm. After removal of the paraffin cover and evaporation of the ammonia, the cores are coated with 3%–4% aminopropyltriethoxysilane (APTES; Sigma Aldrich). This allows for slightly positive cores for deposition of small colloidal gold particles, thus forming what is hence called a seed particle. The seeds are then washed and a 10% gold solution is added to complete the shell. After reaction time, the seeds are washed and re-dispersed in DI water. The seeds are diluted to 0.3–0.5 optical density (OD) at 530 nm (Varian Cary 50 Bio UV-Visible Spectrometer, McKinley Scientific, Sparta, NJ, USA). A sweep of the seeds is performed to optimize the chemical ratio between them, K_2_CO_3_-HAuCl_4_, and formaldehyde (Fisher Scientific). Limiting the concentration of gold in the final reduction step controls the thickness of the gold shell.

### Functionalization of Nanoparticles

The first layer applied to the citrate gold nanoparticles and silica-gold nanoshells was 1-Hexadecanethiol (Sigma Aldrich) dissolved in ethanol. While stirring, 20 mL pure ethanol (Decon Labs) was placed in a beaker with 60 μL 1-Hexadecanethiol being added secondly. The nanoparticles were added to the sample slowly over the next few minutes. The sample was first agitated via shaking and sonication for 60 minutes, and then placed for 12 hours on an orbital rocker (Boekel Scientific, Feasterville, PA, USA) overnight. The sample was spun down, and the pellet was washed twice and resuspended in chloroform (Sigma Aldrich). The second functionalization was the addition of the PC. The stock solution was made by diluting PC in chloroform, and 100 μL were added to the particles after the thiol layer and allowed to set overnight on an orbital rocker. The solutions were transferred to glass tubes and the chloroform evaporated at ambient temperature. After removal of chloroform, PC-coated citrate gold nanoparticles and silica-gold nanoshells were reconstituted in ddH_2_O to 2 OD.

### Nanoparticle Characterization

Nanoparticle maximum absorption wavelengths were obtained using the Varian Cary 50 Bio UV-Visible Spectrometer (McKinley Scientific). Nanoparticle size and zeta potential measurements were obtained using the Zeta-Sizer Nanoseries ZS90 (Malvern Instruments, Worcestshire, UK). Hydrodynamic size based upon Brownian motion was measured in phosphate buffered saline (PBS) solution using DLS (dynamic light scattering). Shape and size were also previously determined using scanning electron microscope (SEM), with the presence of lipids on the particle cores confirmed using a Fourier transform infrared (FTIR) spectroscopy [18].

### Cell Culture

The highly metastatic pancreatic adenocarcinoma S2-VP10 cell line expressing luciferase, a sub-clone of the SUIT-2 pancreatic adenocarcinoma cell line, was obtained from Dr. Michael Hollingsworth (University of Nebraska) [30]. Cells were grown in DMEM with 10% FBS and 1% L-glutamine at 37°C in a humidified incubator.

### Human Pancreatic Cancer Orthotopic Xenograft Mouse Model

Strict adherence to the University of Louisville Institutional Care and Use Committee (IACUC) approved protocol was upheld for the *in vivo* experiments, and the Committee approved this study. Severe combined immunodeficiency (SCID) female mice (Harlan, Indianapolis, IN) received orthotopic pancreatic injections of S2-VP10 metastatic pancreatic adenocarcinoma cells expressing luciferase, resulting in pancreatic tumors within 7 days. The procedure for orthotopic pancreatic cell implantation was previously described [31]. Briefly, mice were anesthetized with isoflurane (≤4% isoflurane for induction and ~1.5% maintenance dose) at 100% O_2_. The left upper abdominal quadrant of the animals was sterilized before making a 1-cm incision. The pancreas was localized using forceps, and the tail of the organ was injected with 1.5×10^5^ cells/30 μL S2-VP10 cells expressing luciferase using a 28-gage needle. Peritoneal leakage of cell solution from pancreatic injection site was minimized by applying a sterile cotton tip applicator for 30 seconds. Organs were returned to normal anatomical position prior to closing the skin and peritoneum using 5–0 Nylon sutures. Animals recovered in a warm area and received liquid acetaminophen for 24 hours post-surgery. Tumor growth was monitored using the AMI-1000X bioluminescence imaging system (Spectral Imaging Instruments, Tucson, AZ). Luciferin (2.5 mg) was given via intraparenteral injection to each mouse 10 min. prior to imaging.

### Intravenous Injection of Nanoparticles

An intravenous (tail vein) injection of 200 μL 2 OD nanoparticle solutions was given to the mice nine days after tumor cell implantation. As there were five mice in each group, the first group received an injection of PC-coated citrate gold nanoparticles; the second group mice received an injection of PC-coated silica-gold nanoshells, and the third group was the negative control (no particle injection). Out of the original number of 19 mice, two mice died after surgical implantation of the orthotopic tumor and two mice died after nanoparticle injection.

### Mouse Euthanasia, Organ Resection, and Histological Processing

Nanoparticles were allowed to circulate for 48 hours before mice were euthanized using CO_2_. The pancreatic tumor, liver, and spleen were removed from each mouse for histological processing. Histology was performed by the University of Louisville Pathology Laboratory (Louisville, KY, USA). Tissues were cut into 4 μm sections and placed onto slides before undergoing a series of ethanol and xylene washes. Cover slips were applied before experimental analysis.

### Nanoparticle Detection using Hyperspectral Imaging

Hyperspectral imaging in combination with dark field microscopy was used to assess nanoparticle uptake and distribution within histology tissue sections. The CytoViva Hyperspectral Imaging System (CytoViva Inc., Auburn, AL, USA) was used for this purpose. This system uses a Dage camera with a microscope with dark field capability. Hyperspectral profiles are acquired using a Pixelfly camera and visualized using ENVI 4.8 software (Exelis Visual Information Solutions, Boulder, CO, USA). To confirm the identity of nanoparticles, spectral libraries were created using z-spectral profiles and compared to the tissue samples. We found that spectral mapping was able to detect and confirm the nanoparticles within tissue samples by using multiple tissue images (> 10) containing either nanoparticles or no nanoparticles. Data analysis was performed with ImageJ. Regions of interest were randomly determined and particles within these regions were counted to determine the concentrations in liver, spleen, and pancreatic tumor tissues. A sufficient number of regions was evaluated to ensure at least 90% accuracy based on stereological analysis.

### Nanoparticle Detection using Silver Enhancement Stain

Slides were placed in cold acetone (Fisher Scientific) for 30 seconds before being placed in 10% formalin buffer (Sigma Aldrich) for 3 minutes. Slides were then washed two times with deionized water and allowed to air dry for 3 minutes. The silver enhancement solution was prepared by mixing 1 mL/slide Silver Enhancement Stain A along with 1 mL/slide Silver Enhancement Stain B (Sigma Aldrich). The two solutions were mixed in a 50 mL tube and vortexed for 10–15 seconds. The combined solution (2 mL) was added to each slide and allowed to react for 6 minutes. After 6 minutes, slides were washed with deionized water. Slides were analyzed using NIS Elements AR and an Accuscope 3032 inverted light microscope. Region of interest (ROI) intensity measurements were recorded for both stained and unstained samples. Intensity value of unstained ROI was subtracted from stained ROI to obtain a net ROI intensity.

### Statistical Analysis

Analyses used the two-tailed Student’s t-test with a significance level of 0.05. Statistically significant results are illustrated with an asterisk (*) in the Results.

## Results

### Nanoparticle Synthesis and Characterization

**Fig 1:**
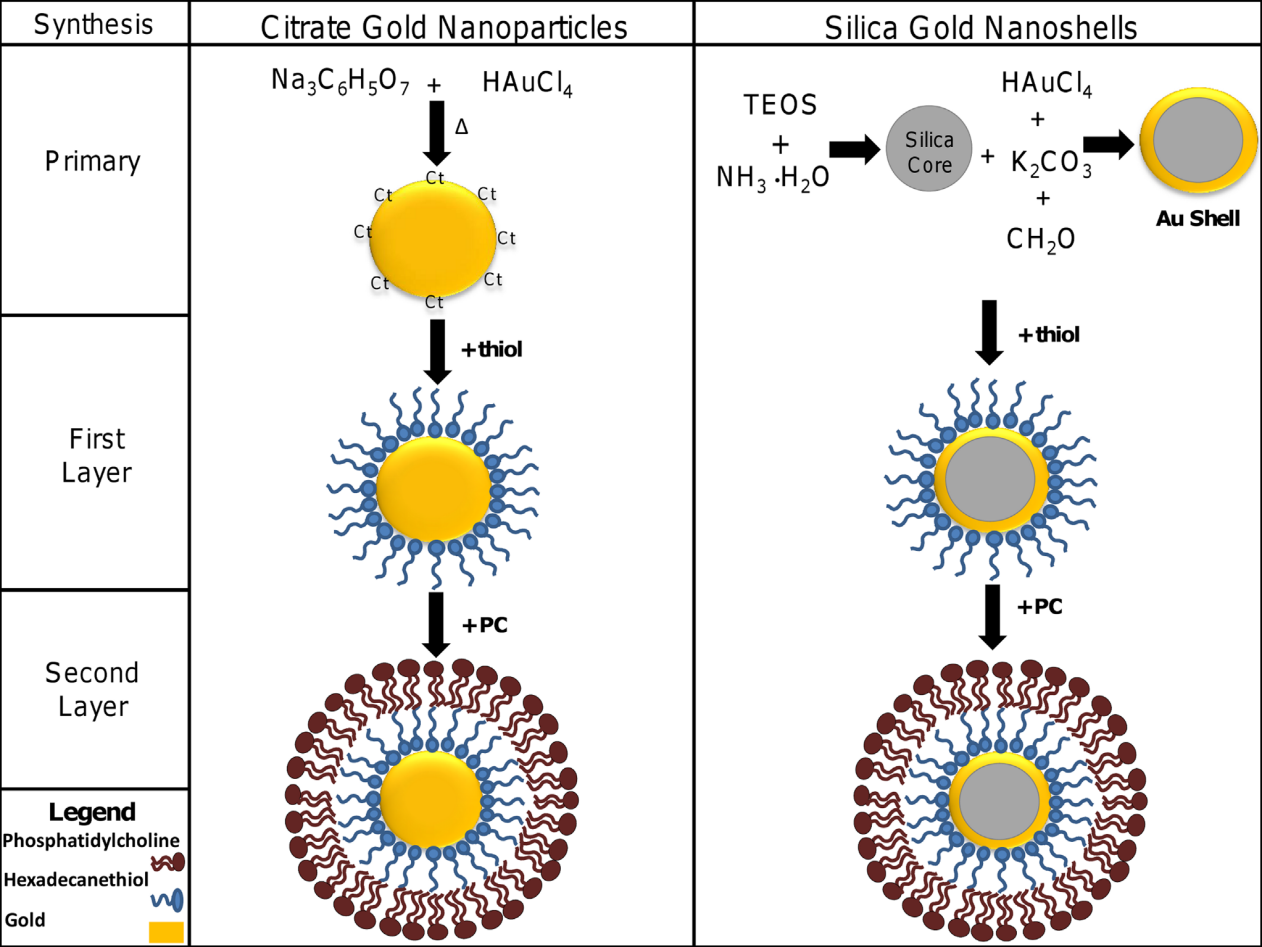
Synthesis of nanoparticles was completed using methods described previously. Briefly, citrate gold nanoparticles were synthesized by the reduction of chloroauric acid by sodium citrate. The citrate is removed from the surface by the addition of hexadecanethiol, which is used for the conjugation of phosphatidylcholine. Silica-gold nanoparticles were synthesized by first creating silica cores before adding ultra-small colloidal gold nanoparticles onto the surface of the cores to create a thin layer of gold through a reduction process. The same modifications of hexadecanethiol and phosphatidylcholine were applied to the silica-gold nanoshells. TEOS: tetraethyl-orthosilicate.

The addition of a PC layer to citrate gold nanoparticles and silica-gold nanoshells was accomplished using a layering process in which the charged head group of hexadecanethiol binds to the nanoparticle (Fig 1), resulting in hydrophobic nanoparticles with hydrocarbon chains pointed outward towards the surrounding environment. The addition of PC to the hexadecanethiol-coated nanoparticles binds tail-to-tail creating water-soluble nanoparticles containing an inner layer suitable for hydrophobic drug loading.

**Fig 2:**
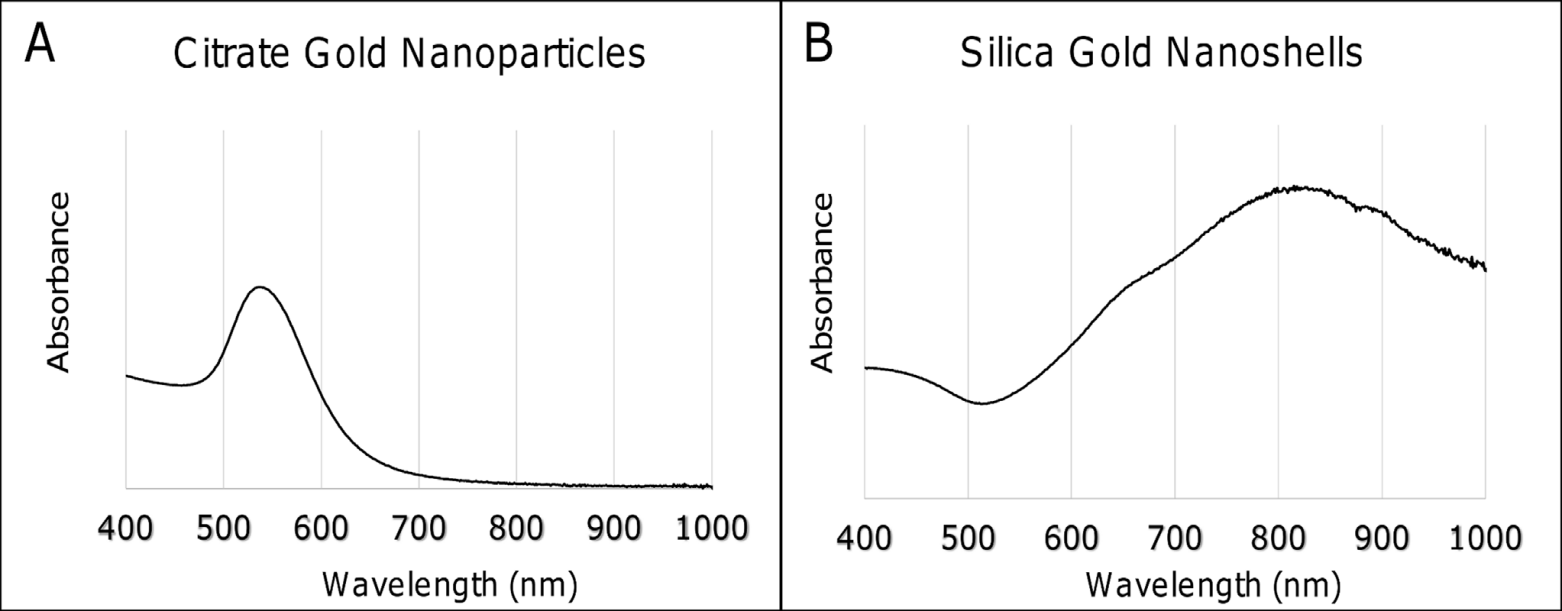
Spectra of nanoparticles were obtained through UV-Visible spectroscopy. (A) Citrate gold nanoparticles displayed a maximum absorption at 540 nm. (B) Silica-gold nanoshells displayed a maximum absorption at 830 nm.

Optical measurements of functionalized citrate gold nanoparticles and silica-gold nanoshells confirmed nanoparticle identity and functionalization (Fig 2). Colloidal gold nanoparticles exhibit UV-Vis maximum absorbance values ranging between 510–550 nm, in agreement with the PC-coated nanoparticles experiencing an optimal peak at 540 nm (Fig 2A). In comparison, previously synthesized PEGylated citrate gold nanoparticles displayed a maximum wavelength of 533 nm [18]. Maximum absorbance of silica-gold nanoparticles is based upon the diameter of the silica core and size of the gold shell surrounding the core, with smaller shells producing larger wavelengths. Silica-gold nanoshells synthesized in this study displayed a maximum absorbance of 835 nm that is similar to silica gold nanoparticles with ~110nm diameter silica cores and ~10–15 nm gold coating (Fig 2B). In comparison, previously synthesized PEGylated silica-gold nanoshells displayed a maximum absorbance of 820 nm [18]. We have previously shown that silica-gold nanoparticles commonly possess wavelengths ranging from 820–860 nm based upon sizing and surface modifications [18].

**Fig 3:**
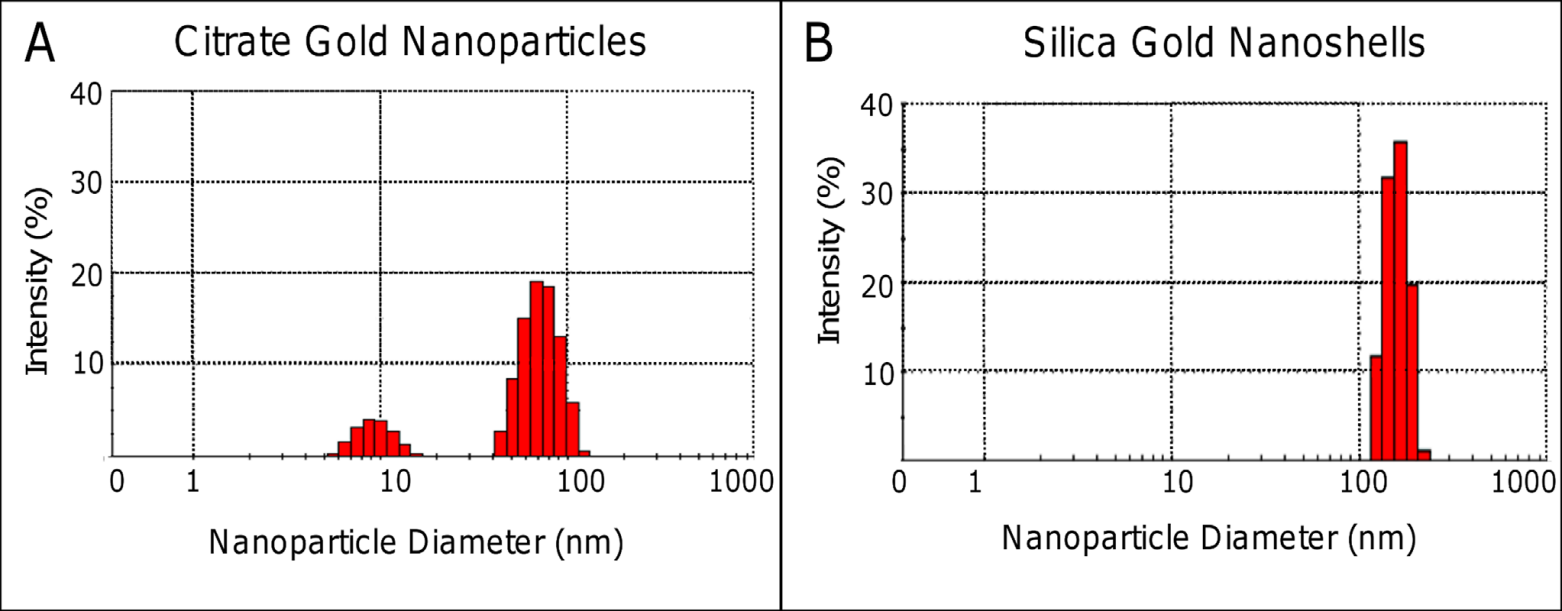
Hydrodynamic size of nanoparticles was measured using DLS. (A) The diameter of PC-coated citrate gold nanoparticles was 82.3 ± 9.4 nm, while that of PC-coated silica gold nanoparticles was 144.13 ± 11.7 nm. Smaller peak shown for citrate gold corresponds to smaller components formed during the synthesis process. Overall intensity values shown do not total 100% due to some agglomerated particles, whose intensities were not included in the figure.

**Fig 4:**
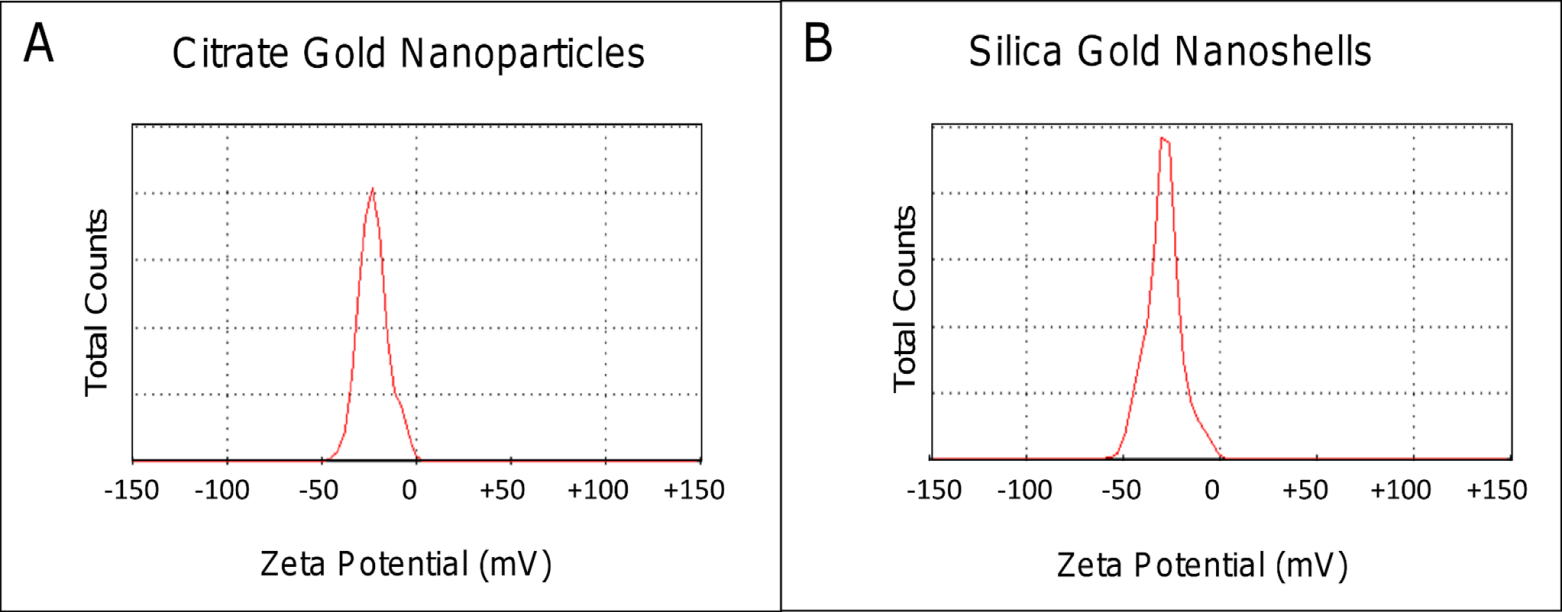
The surface charge of nanoparticles was measured using the Zetasizer. The surface charge, or zeta potential, of these nanoparticles displayed similar cationic surfaces with (A) the PC-coated citrate gold nanoparticles possessing a zeta potential of -23 mV. The PC-coated silica-gold nanoshells were slightly more cationic at -31 mV.

Further characterization elucidated information regarding hydrodynamic size and surface charge (Fig 3). The hydrodynamic diameter has a crucial impact on deposition patterns *in vivo* as a thin electric dipole layer of the solvent adheres to the surface of the nanoparticles as they move through a liquid medium under the effects of Brownian motion. The size determined through DLS was greater than the sizing information obtained visually by SEM, which is a common occurrence [32]. The PC-coated citrate gold nanoparticles had an average DLS size of 82.3 ± 9.4 nm (Fig 3A), while PC-coated silica-gold nanoparticles had an average size of 144.13 ± 11.7 nm (Fig 3B). These DLS sizes can be contrasted with previously synthesized PEGylated citrate gold nanoparticles and silica-gold nanoshells exhibiting sizes of 82.81±13.4 nm and 161.82±12.2 nm, respectively [18]. The size difference between citrate gold and gold nanoshells is expected to have an impact on diffusivity within *in vivo* tumor tissue, in accordance with previous observations in 3D cell culture [18]. The surface charge showed similar zeta potentials between the citrate gold nanoparticles and silica-gold nanoshells of -23 mV (Fig 4A) and -31 mV (Fig 4B), respectively.

### Creation of Nanoparticle Spectral Libraries

**Fig 5:**
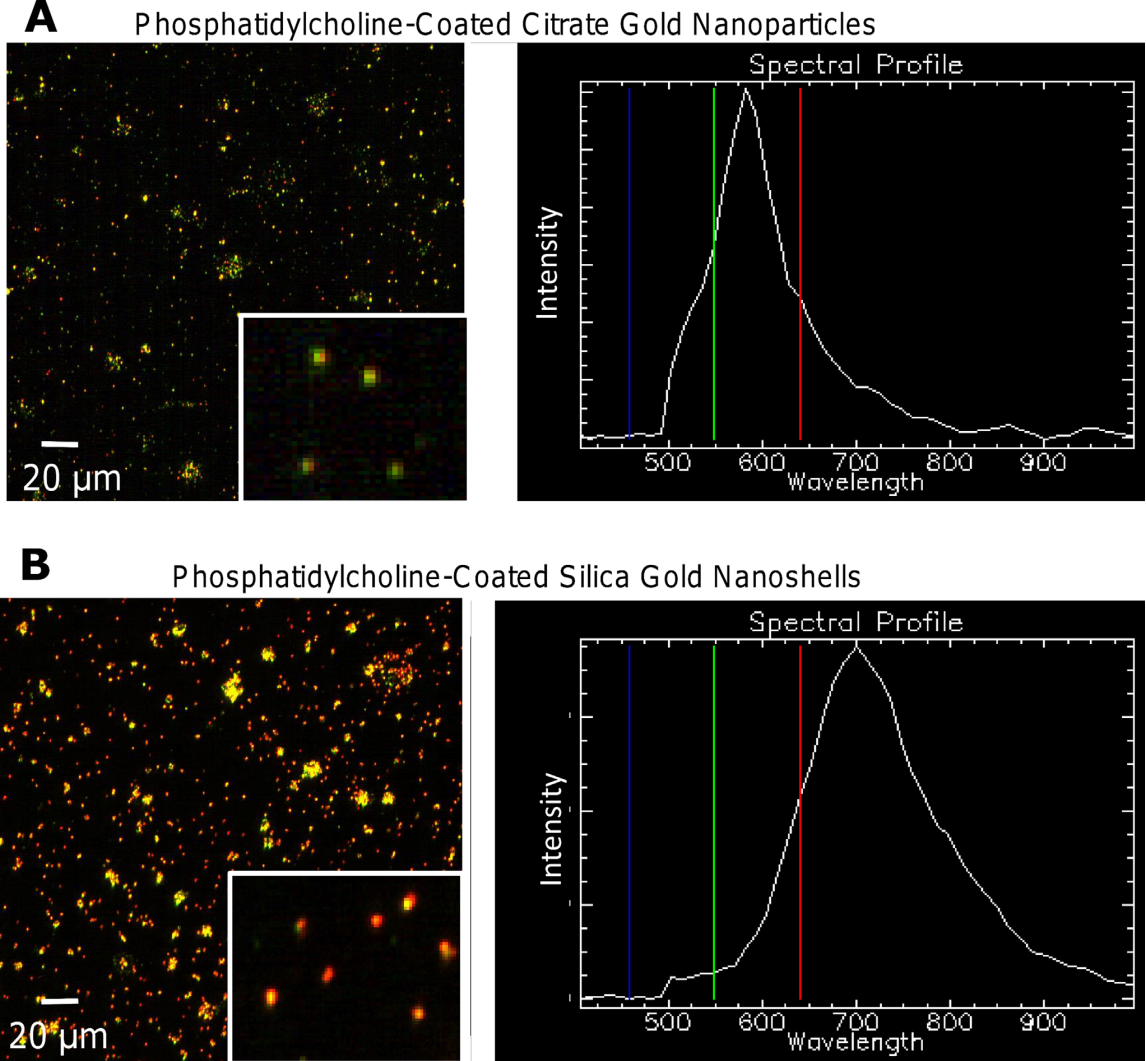
Hyperspectral imaging of PC-coated citrate gold nanoparticles and silica-gold nanoshells exhibited unique spectral profiles. (A) Citrate gold nanoparticles were primarily green, with a spectral profile indicating a maximum wavelength of 570 nm. (B) Silica-gold nanoshells displayed an abundance of red and yellow nanoparticles, with a spectral profile maximum of 700 nm. While the actual maximum wavelength of silica-gold nanoshells in solution is between 820–860 nm, hyperspectral imaging is known to underestimate entities containing higher wavelength maxima. Insets illustrate the current capability to visualize single particles with this system.

Before nanoparticles were detected in tissue samples from mice, spectral libraries were created of the PC-coated citrate gold nanoparticles and silica-gold nanoshells that could be later matched to the images for detection (Fig 5). The spectral libraries were synthesized from samples of both types of nanoparticles in solution. Visible color changes from green to yellow to red illustrated the shift of the maximum absorbance wavelength of the nanoparticles. For PC-coated citrate gold nanoparticles, the majority of nanoparticles in solution exhibited green color indicating the particles processed maximum absorbance values within the lower range (500–600 nm) (Fig 5A). This was confirmed through the z-profile spectra mapping showing a maximum band ~550–570 nm. The solution of PC-coated silica-gold nanoshells contained a significant concentration of red nanoparticles, indicative of absorbance at higher wavelengths (>700 nm) (Fig 5B). As nanoshells exhibit wavelengths higher than colloidal gold nanoshells, this also helped to confirm the identity of the nanoparticles.

### Evaluation of Nanoparticle Uptake and Distribution

**Fig 6:**
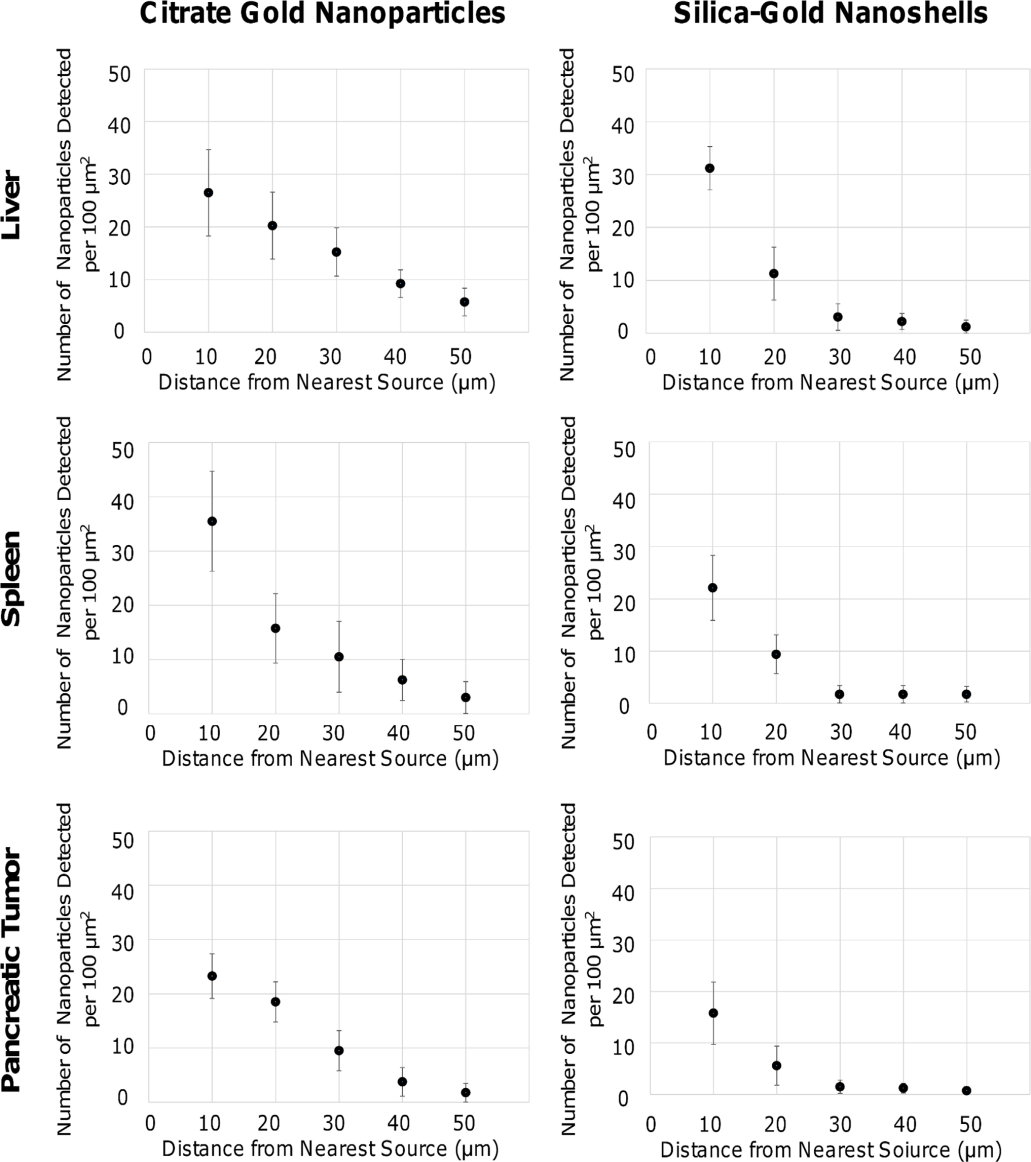
Citrate gold nanoparticle and silica-gold nanoshell penetration was measured in liver, spleen, and pancreatic tumor tissue samples. Distances from the nearest source were measured using hyperspectral imaging. In the liver and pancreatic tumor, citrate gold nanoparticles had a linear diffusion pattern, while the spleen had an exponential decline. Silica gold nanoshells had an exponential decline, suggesting limited diffusivity in all three tissue types. Citrate gold nanoparticles had highest extravasation close to their sources in the spleen while for silica gold nanoshells extravasation was highest in the liver. All error bars denote standard deviation (n = 3).

The creation of spectral libraries of PC-coated nanoparticles enables nanoparticle detection in tissue samples. After nanoparticles extravasate from vasculature, they diffuse through the tissue composed of cells and ECM. In some tissues, extravasation sources residing relatively close to each other can provide a more homogenous layout of source points. Nanoparticle counts were obtained in the vicinity of source points in histology sections to determine the uptake and distribution in sections of liver, spleen, and orthotopic pancreatic tumor (Fig 6).

For citrate gold nanoparticles, the liver and pancreatic tumor on average exhibited a linear decline in nanoparticle concentration from the nearest source, with 26.5 ± 8.2 and 23.3 ± 4.1 particles/100μm^2^, respectively, within 10 μm of the source and few nanoparticles penetrating beyond 50 μm (Fig 6). The spleen displayed on average a higher uptake of 35.5 ± 9.3 particles/100μm^2^ within 10 μm followed by an exponentially decaying penetration pattern, also limited to ~50 μm (Fig 6).

Silica-gold nanoshell were uptaken the most within the liver showing on average 31.1 ± 4.1 particles/100μm^2^ within 10 μm of the nearest source, with penetration hindered beyond 30 μm (Fig 6). The spleen and pancreatic tumor also exhibited limited penetration beyond 30 μm (Fig 6), with uptake of 22.1 ± 6.2 and 15.8 ± 6.1 particles/100μm^2^, respectively, within 10 μm. While the penetration of citrate gold nanoparticles suggests linear diffusion, the nanoshells displayed more of a decaying exponential pattern further implying limited diffusivity. We hypothesize that the limited diffusivity of nanoshells in the liver, spleen, and pancreatic tumor is size-dependent, thus larger particles (>150 nm) would experience even greater diffusion limitations.

### Nanoparticle Accumulation in Liver, Spleen, and Pancreatic Tumor

**Fig 7:**
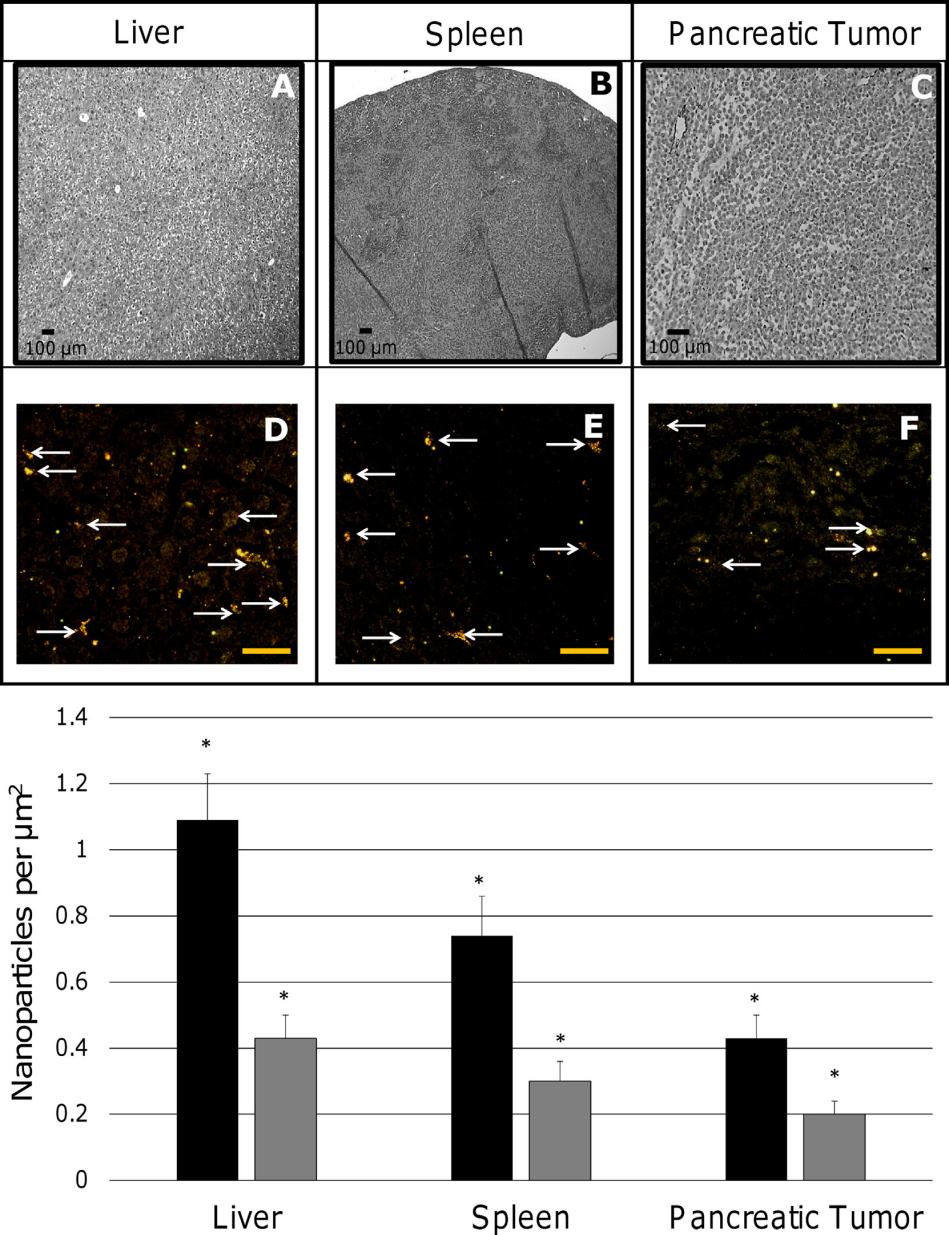
Citrate gold nanoparticle and silica gold nanoshell concentrations within liver, spleen, and pancreatic tumor showed differential uptake. Bright-field visualization of tissue structure for (A) liver, (B), spleen, and (C) pancreatic tumor used to locate potential nanoparticle extravasation sites. Hyperspectral imaging of nanoparticles in combination with dark field microscopy highlights the density of extravasation sites that may affect the uptake of nanoparticles in these tissues (bars, 25 μm): (D) The liver provides numerous extravasation sites; (E) the spleen also has numerous extravasation sites, albeit to a lesser extent compared to the liver; (F) the pancreatic solid tumor has fewer sites, leading to diminished nanoparticle uptake. Bottom graph: The liver exhibited highest concentration of either citrate gold nanoparticles (black bars) or silica gold nanoshells (grey bars), followed by the spleen and the pancreatic tumor. Statistical significance is signified by (*) with a p-value < 0.05. Error bars denote standard deviation.

Accumulation of nanoparticles is a function of vasculature of an organ or tissue. The liver and spleen with similar patterns of highly vascularized tissue are expected to present a higher number of nanoparticle source points, while the pancreatic tumor with a more heterogeneous vascular pattern would have less. We first used bright-field to first locate potential extravasation sites for each tissue type (Fig 7A–7C), and then switched to dark-field microscopy for analysis. Dark-field tissue sections were examined to evaluate extravasation sites within the tissue (Fig 7D–7F). Some regions of pancreatic tumor may contain large quantities of sprouting vessels due to sporadic angiogenesis, while other regions experience diminished vascular density with possibly few vessels supporting large volumes of tissue. This heterogeneity may result in tumor tissue becoming hypoxic and necrotic, with correspondingly restrained nanoparticle accumulation in these regions.

The average concentration of citrate gold nanoparticles was highest in the liver (1.09 ± 0.14 nanoparticles per μm^2^), as compared to the spleen (0.74 ± 0.12 nanoparticles per μm^2^) and pancreatic tumor (0.43 ± 0.07 nanoparticles per μm^2^) (Fig 7, **graph**). In contrast, the average concentration of silica gold nanoshells was 0.43 ± 0.07 nanoparticles per μm^2^ in the liver, 0.30 ± 0.06 nanoparticles per μm^2^ in the spleen, and 0.20 ± 0.04 nanoparticles per μm^2^ in the pancreatic tumor. Using the two-tailed student t-test, statistical significance is shown (all p-values < 0.05). The concentration of nanoparticles in normal pancreas was observed to be lower yet more homogeneously distributed than in tumor regions.

**Fig 8:**
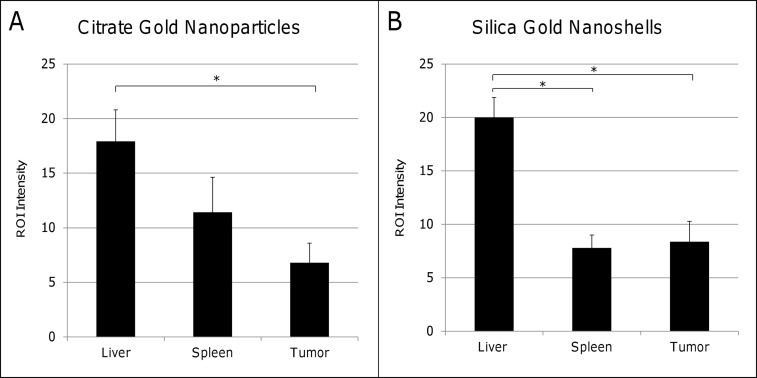
Silver enhancement staining confirmed the presence of nanoparticles within liver, spleen, and pancreatic tumor tissue sections. (A) The liver displayed higher ROI intensity values for citrate gold nanoparticles in comparison to tumor sections. (B) The liver also displayed higher intensity ROI values for silica gold nanoshells in comparison to both spleen and tumor sections. Statistical significance is signified by (*) with a p-value < 0.05. Error bars denote standard deviation.

Nanoparticle presence in the tissues was independently assessed using silver enhancement staining (Fig 8). The liver displayed highest staining for both citrate gold nanoparticles and silica gold nanoshells, with the pancreatic tumor having significantly lower staining intensity. This intensity depends not only on particle number but also on particle size and shape, and thus provides a qualitative assessment.

## Discussion

We used hyperspectral imaging to detect phosphatidylcholine-coated citrate gold nanoparticles and silica-gold nanoshells in tissue excised from mice *in vivo*. In particular, phosphatidylcholine (PC) is the most abundant phospholipid found in cellular membranes and thought to decrease possible immunogenicity [33]. The addition of PC was previously shown to significantly reduce associated cytotoxicity and particle aggregation, while enhancing passive targeting capabilities [34]. In previous work, we observed enhanced diffusivity of PC-coated nanoparticles in 3D cell cultures, with superior uptake and penetration in comparison to PEGylated nanoparticles [18]. PEG is currently the most widely employed FDA-approved polymeric platform used in the synthesis of medicinal nanoparticles for treating various cancers and related diseases, including Genexol-RM for metastatic breast cancer, Oncaspar for acute lymphoblastic leukemia, and Neulasta for chemotherapy-associated neutropenia [35]. While PEGylation is effective in enhancing drug delivery, multiple adverse effects have been associated with the use of PEG including possible immunogenicity, which is still debated [36]. The evaluation of PC-coated nanoparticles *in vivo* may longer term enable an alternative therapeutic option. We note that the only FDA-approved nanotherapeutic currently available for pancreatic cancer is Abraxane, an albumin-bound form of paclitaxel [37].

In this study, the formation of a bilayer membrane around nanoparticles was achieved by first adding hexadecanethiol, which displaced the outer coating of citrate and formed a hydrophobic nanoparticle (Fig 1). Through the addition of PC, the hydrophobic tails of the hexadecanethiol interact with the hydrocarbon chains of PC to form the membrane layer. The hydrophobic region between hexadecanethiol and PC can potentially be utilized for entrapping hydrophobic drugs [38], resulting in enhanced bioavailability. In addition, PC-coated silica-gold nanoshells exhibit wavelengths within the near infrared (nIR) region, making them potential candidates for photothermal ablation therapy as well as diagnostic imaging through multispectral optoacoustic tomography (MSOT) [39–42]. Recently, specific uptake of hybrid iron-oxide core gold-shell nanoparticles by pancreatic cells in photothermal therapy has been demonstrated. Cells exposed to nanoparticles and laser irradiation produced dose-dependent temperature increases and a reduction in cell proliferation, suggesting that photothermal ablation may be of use in the treatment of pancreatic cancer [43].

Detection of nanoparticles in tissue samples has been extensively accomplished using electron microscopy techniques (TEM, SEM, STEM) [44], yet newer methodologies present opportunities. Additional studies have utilized confocal microscopy to examine the translocation of nanoparticles in co-culture cancer models [45]. While these models can detect nanoparticles, they lack the ability to confirm the presence of nanoparticles in tissue based upon spectral mapping. Hyperspectral imaging of nanoparticles in solution demonstrates the feasibility of determining the spatial location, agglomeration status, wavelength differentiation, and partial size determination [21, 46]. Using hyperspectral imaging, nanoparticles can be further analyzed and characterized to determine properties such as surface modifications.

In this study, we used hyperspectral imaging to assess the uptake and distribution of gold nanoparticles within tissue samples. We note that nanoparticle agglomeration can distort the results when attempting to assess nanoparticle concentrations within regions of interest. Consequently, numerical data gained from this method should be considered approximate and not absolute. Also, nanoparticle spectral profiles can be altered by factors including agglomeration status, backscatter from other sources, and other mechanisms. While the citrate gold nanoparticles exhibited similar spectra to those adapted from UV-Vis spectroscopy, the silica-gold nanoshells displayed a lower maxima (Fig 2). Such small changes are considered normal. Another common occurrence in detecting nanoparticles stems from the use of dense tissue samples, in which light scattering from different tissue components may hinder proper view. For this reason, spectral matching should be mapped for each image to computationally extract the nanoparticles, providing a clear depiction of which positive pixels are nanoparticles as compared to other tissue elements.

The spatial distribution of vessels within organs can cause some data distortion as vascular source points in mice reside within close proximity of each other [47]; nanoparticles extravasated from a particular source point may thus overlap with those extravasated from a nearby source. This was considered when counting the nanoparticles and was a reason to limit the distance of evaluation to 50 μm, which is the typical inter-vessel distance in the murine liver [48].

Recently, we have evaluated uptake and penetration of functionalized nanoparticles in avascular 3D cell cultures [18]. Diffusion is both size and surface modification dependent, shown by analyzing the penetration of citrate gold nanoparticles and silica gold nanoshells (size difference ~80 nm) based on functionalization with TL and PC, or TL, PC, and HDL, and finding that coating with PC or HDL increased uptake and penetration [18]. The results herein show that PC-coated citrate gold nanoparticles exhibit a quasi-linear diffusion pattern *in vivo* similar to the 3D cell cultures (Fig 6). In contrast, PC-coated silica gold nanoshells experienced a steeper decline in concentration from the nearest source point (Fig 6) compared to that observed in 3D cell culture [18]. Further, the particle uptake observed within 10 μm of the nearest source in the pancreatic tumor was on average 67% lower than observed with these same cells *in vitro*. While 3D cell cultures mimic the morphological structure of tissue *in vivo*, tissue found within live subjects is more complex. This tissue is composed of a fully developed extracellular matrix and multiple cell types (including cells of the immune system) that are in contact with the blood supply and the lymphatic system, which may affect the deposition patterns and uptake of nanoparticles beyond the effects from 3D space. The extravasation *in vivo* is also expected to be hindered by interstitial fluid pressure within tumor tissue as has been shown in experimental as well as modeling studies [6, 11, 49, 50]. The elevated concentration of nanoparticles in the liver and spleen highlights the sequestering by the RES (Fig 7). Overall, the smaller citrate gold nanoparticles exhibited enhanced accumulation compared to the silica gold nanoshells. This difference can be primarily attributed to their smaller size. Nevertheless, the hypo-vascularization in the pancreatic tumor tissue can significantly hinder nanoparticle uptake regardless of size (Fig 7, **graph**).

We note that, in general, orthotopic mouse models do not create a similar stromal reaction within the tumor like that of human pancreatic adenocarcinoma. Consequently, such models may be more responsive to chemotherapeutic agents. In contrast, Kras genetically engineered mouse models (e.g., KPC-mouse) are known to better recapitulate the stromal component of human pancreatic adenocarcinoma. Further, orthotopic mouse models using S2-VP10 cells may grow very fast, creating centrally necrotic tumors—such necrotic tumors are seldom seen in humans.

This study can be continued by examining the drug loading efficiency of the PC-coated nanoparticles. Hydrophobic drugs could be loaded into the hydrophobic region between the hexadecanethiol and PC, and thus facilitate their delivery through the hydrophilic tissue environment. To enhance local release, one would require the use of gold particles which absorb light in a region transparent to tissue, such as near infrared (nIR) absorbing gold nanoparticles (gold silica nanoshells, nanorods, or gold-sulfide aggregate nanoparticles) [51]. Drug release would then be mediated by heating of the surface (using the correct amount of energy via light dosing) to allow release of surface bound molecules. This method would minimize damage to surrounding tissue. Further, the nIR wavelength associated with the gold silica nanoshells in this study makes them potential candidates for photothermal ablation therapy. Citrate gold colloid based particles should not be used for thermal ablation as the wavelength of light (~540 nm) used for activation will cause harm due to absorption of energy by the tissue. Nevertheless, citrate gold NPs may be easier for designing a layered system for drug transport modeling studies [38]. Further, evaluation of nanoparticle distribution and uptake *in vivo* at later timepoints may enable evaluation at metastatic sites; here, as a first step, we focus on the uptake of the nanoparticles at the primary site. Finally, by analyzing diffusivity and distribution patterns of these nanoparticles *in vitro* and *in vivo*, mathematical modeling could be applied to help design and project treatment outcomes [12, 50, 52–55].
